# Dynamics of p53: A Master Decider of Cell Fate

**DOI:** 10.3390/genes8020066

**Published:** 2017-02-09

**Authors:** Qingyin Luo, Jill M. Beaver, Yuan Liu, Zunzhen Zhang

**Affiliations:** 1 Department of Environmental Health and Occupational Medicine, Sichuan University West China School of Public Health, Chengdu 610041, China; cherry12112009@163.com; 2 College of Food Science, Sichuan Agricultural University, Yaan 625014, China; 3 Biochemistry Ph.D. Program, Florida International University, Miami, FL 33199, USA; jbeav004@fiu.edu; 4 Department of Chemistry and Biochemistry, Florida International University, Miami, FL 33199, USA; 5 Biomolecular Sciences Institute, School of Integrated Sciences and Humanity, Florida International University, Miami, FL 33199, USA

**Keywords:** p53 dynamics, cell fate decision, cell signaling network

## Abstract

Cellular stress-induced temporal alterations—i.e., dynamics—are typically exemplified by the dynamics of p53 that serve as a master to determine cell fate. p53 dynamics were initially identified as the variations of p53 protein levels. However, a growing number of studies have shown that p53 dynamics are also manifested in variations in the activity, spatial location, and posttranslational modifications of p53 proteins, as well as the interplay among all p53 dynamical features. These are essential in determining a specific outcome of cell fate. In this review, we discuss the importance of the multifaceted features of p53 dynamics and their roles in the cell fate decision process, as well as their potential applications in p53-based cancer therapy. The review provides new insights into p53 signaling pathways and their potentials in the development of new strategies in p53-based cancer therapy.

## 1. Introduction

Cells are constantly bombarded by a variety of endogenous and environmental stress that results in cellular damage. This usually leads to cell cycle arrest allowing the damage to be repaired by cellular repair mechanisms. However, in an occasion of severe damage, cells have to directly activate a “suicide” apoptotic death program to induce cell death, preventing initiation of oncogenic processes. Thus, cells have two possible fates—to survive or die. Failure in making the right decision in determining cell fate can ultimately lead to the development of diseases such as cancer. The cell fate decision machinery is composed of multiple complex signaling pathways, in which p53, a key tumor suppressor, plays a central role in coordinating the multiple cellular signaling pathways as well as determining cell fate [1].

As a transcription factor, p53 is activated by various types of stimuli. This selectively regulates the transcription of p53 target genes that subsequently mediate cellular responses to stress. In the past two decades, hundreds of p53 target genes have been identified. These include the cyclin-dependent kinase inhibitor *P21* gene and pro-apoptotic genes such as *BAX*, *BAK*, *PUMA*, and *P53AIP1* [2,3,4]. The proteins encoded by these genes are involved in inhibition of cell proliferation or activation of apoptosis. In addition, several microRNAs, most notably the miR-34 family, that are involved in cell cycle arrest, and large intergenic noncoding RNAs (lincRNAs) that are involved in transcription repression, have also recently been identified as p53 target genes [5,6]. While the list of p53 target genes keeps growing, the multiple components of the list build up a p53 signaling network providing massive information about how p53 may promote cellular growth arrest or apoptosis. However, this raises a question as to whether the biological function of p53 can be completely elucidated by just simply glimpsing its static network. In fact, similar to our human body that uses its central nervous system to transduce signals and regulate and coordinate the movement of the entire body dynamically, p53 also functions in a dynamic manner and its dynamics can also serve as a central component of its signaling network to integrate, coordinate, and regulate cellular stress responses in a dynamic manner.

**Figure 1:**
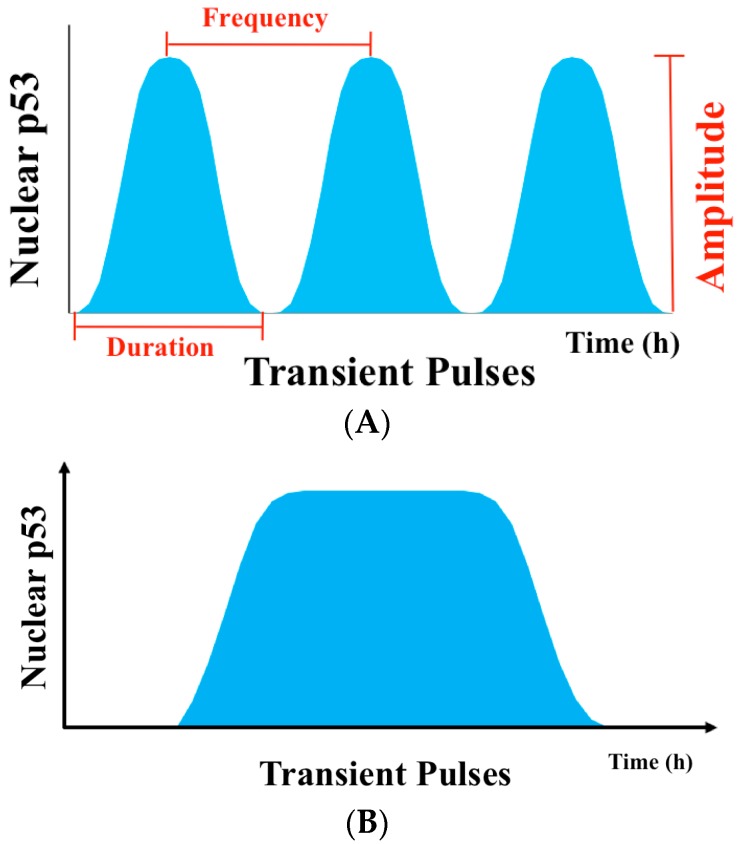
p53 dynamics in damage response. (**A**) p53 dynamics can be measured at the levels of frequency, amplitude, and duration; (**B**) A single prolonged pulses of p53 induced by UV radiation.

**Figure 2:**
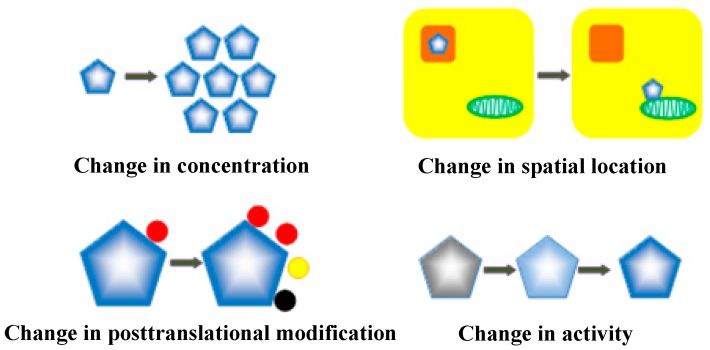
p53 dynamics. p53 dynamics include the changes of p53 protein concentration, activity, localization, or modifications that can be measured over time.

In this review, we discuss the progress in an emerging new field in cell biology that explores a p53-mediated cellular signaling network via the dynamics of key signaling molecules in the network [7,8], where the type and quantity of a stimulus can result in a specific pattern of p53 dynamics. This is manifested by activation of a group of p53 target genes and the resulting biological outcome in cells. Since p53 dynamic changes have been mainly observed in the level of p53 protein, which can be quantitatively measured over time (Figure 1), “p53 dynamics” usually refers to the dynamic changes of p53 protein level. However, other types of p53 dynamic changes that include dynamics in its subcellular location, activity, and posttranslational modifications, and the interplay among its different dynamic features, should also be essentially important in mediating a stimulus-specific response [7,9]. In this review, we initially discuss p53 dynamics as a sum of the dynamic changes of its protein level or concentration, activity, subcellular localization, and posttranslational modifications (Figure 2). We then discuss the dynamics of p53 that underlies its cellular signaling network and the biological functions of the network. Finally, we propose a hypothetical model to illustrate the central roles of p53 dynamics in determining cell fate and discuss the potential application of p53 dynamics in p53-based cancer therapy.

## 2. Pulsatile p53 Dynamics of Protein Level

### 2.1. The Dynamics of p53 in Single Cells

In the 1990s, it was observed that treatment of rat neuronal precursors with epidermal growth factor (EGF) and neural growth factor (NGF) can lead to cell proliferation and differentiation, respectively. Further investigation revealed that both stimuli activated the same protein kinase, the extracellular signal-regulated kinase (ERK), with a distinct temporal feature—i.e., EGF induces a transient ERK activation—whereas NGF triggers a sustained ERK activation [10,11,12]. This suggests that the temporal features of ERK activation—i.e., its dynamics—play a pivotal role in ERK-mediated cell signaling. This concept was later used to propose a model for the roles of p53 in mediating cell signal transduction while it receives and transmits cellular signals via its dynamic features. The model is supported by a number of studies. For example, it has been shown that the total p53 protein level in PC12 cells was initially increased by gamma irradiation (IR), and then decreased in a series of damped oscillations [13]. The oscillations in p53 protein level were also observed in a variety of cell lines as well as in a transgenic mouse model wherein the firefly luciferase gene expression was dependent on the p53-responsive P2 promoter [13,14]. Employing a fluorescence-tagged p53 protein and high-resolution time-lapse imaging technology that can detect the protein level in a single cell, it was found that the p53 protein level in a single PC12 cell actually exhibited a series of undamped pulses with a fixed amplitude and duration (Figure 1B) [15]. Thus, the observed damped p53 oscillations in a population of cells are in fact the average of total oscillations of a group of unsynchronized cells with reduced pulse signals over time [7]. Thus, to determine cellular p53 dynamics accurately, it is essentially important to study p53 dynamics in a single cell as this allows accurate determination of the dynamic patterns of p53 protein level induced by different stimuli in each individual cell. For example, in individual PC12 cells, low doses of ionizing radiation (IR) lead to a series of transient pulses of p53 protein level with fixed amplitude and duration. However, exposure to high doses (from 2.5 to 10 Gy) of IR or long time periods of exposure to IR (from 2 to 24 h) only increased the number of p53 pulses without affecting their amplitude and duration (Figure 1A) [16]. In contrast, ultraviolet (UV) radiation triggers a single sustained pulse of p53 protein level in a dose and/or time-dependent manner [17] (Figure 1B). The observation indicates that only p53 dynamics at a single cell level can accurately reflect its function in governing p53-mediated cell signaling, i.e., different stimuli can induce unique p53 dynamical patterns/features in each individual cell. This notion has been further supported by a recent study that has identified significant variations in p53 dynamics in different individual colon cancer cells, which determine different fates of cancer cells in response to a chemotherapeutic drug [18].

Furthermore, several studies support the notion that cells can translate different p53 dynamical patterns into a specific type of downstream cellular responses. It has been found that IR-induced transient p53 pulses are associated with activation of pro-cell cycle arrest genes that result in cell-cycle arrest, whereas prolonged p53 pulses induced by UV are involved in upregulation of pro-apoptotic genes that can lead to apoptosis [17]. This is further supported by a study from the Lahav group [19] showing that DNA damage induced by moderate doses of IR triggered transient pulses of p53. This, in turn arrests the cell cycle, facilitating damage repair and cell survival. On the other hand, sustainment of a high level of p53 protein with a small compound, Nutlin-3, that switches p53 dynamics from a pulsed mode to a sustained mode, leads to cell senescence [19]. The results suggest that a specific dynamic pattern of p53 results in a specific cellular outcome. Thus, different patterns of p53 dynamics can be “translated” into different downstream responses and cellular outcomes by the downstream proteins in the p53 signaling pathway.

### 2.2. The Role of p53 Dynamics in Making Cell Fate Decisions

#### 2.2.1. How Can Pulsatile p53 Dynamics Be Generated?

As a transcription factor, p53 can activate pro-cell cycle arrest and pro-apoptotic genes as well as induce the expression of genes that regulate its own gene expression and the stability of its protein. This results in the formation of a feedback loop leading to the pulsatile dynamics of p53 protein level.

**Figure 3:**
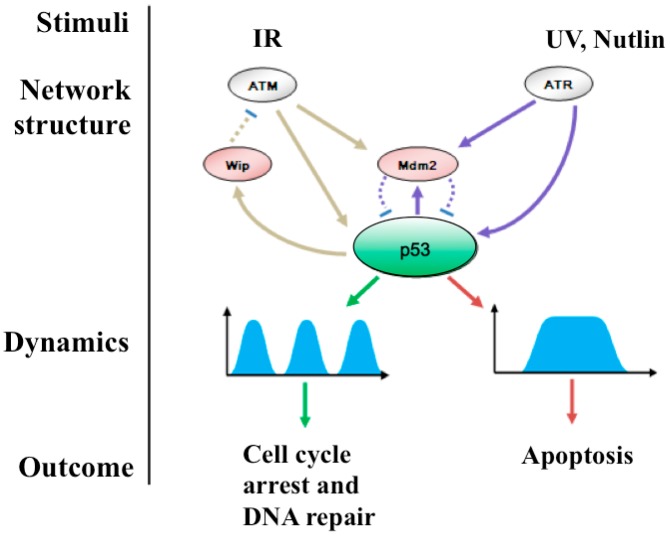
Activation of p53 governs the patterns of p53 dynamics. p53 can be activated by DNA double strand breaks (DSBs) induced by IR or single DNA strand breaks (SSBs) induced by UV radiation or nutlin. p53 can be activated by DSBs. This is mediated by the ATM-p53-Wip loop negative feedback loop. Activation of p53 by SSBs is mediated by the ATR-p53-Mdm2 negative feedback loop. These can subsequently lead to a series of transient pulses or a prolonged pulse, which in turn results in cell cycle arrest, cell recovery or apoptosis.

The first identified feedback loop in the p53 network is the p53 mouse double minute 2 (Mdm2) negative feedback loop. Discovered in 1992, Mdm2 was initially proposed as an oncoprotein because it functions as a p53-specific E3 ubiquitin ligase that promotes ubiquitination and subsequent proteasomal degradation of p53 [20]. Later it was found that the expression of Mdm2 is transcriptionally regulated by p53 [21,22]. The relationship between p53 and Mdm2 has been further elucidated by a computational model and is defined as a negative-feedback loop, where p53 increases the expression of Mdm2, and this, in turn promotes the degradation of p53 protein [14]. The p53-Mdm2 feedback loop leads to oscillations with repeated increases and decreases in cellular p53 protein level building up the basis of p53 dynamics. The existence of p53-Mdm2 negative feedback is further supported by numerous studies [14,23,24] revealing that the dynamics of a signaling molecule is constructed by a feedback loop formed between the components of the cellular signaling pathway. Although the p53-Mdm2 negative feedback loop can serve as a basis of p53 dynamics, it is still too simple for explaining the complex responses within the p53 signaling network [22]. In addition, activation of p53 requires distinct inducers in response to different types of stimuli. To address the issue, several p53 upstream genes were later added to the basic p53-Mdm2 negative feedback loop [25]. Based on this, several advanced models of p53 feedback loops were established. Among them, the negative feedback loops of ataxia telangiectasia mutated (ATM)-p53-wip-Mdm2 loop and ataxia-telangiectasia and Rad3 kinase (ATR)-p53-Mdm2 are two popular ones (Figure 3) [8]. Besides the p53-Mdm2 feedback loop, two additional p53-ubiquitin ligases, Cop1 and Pirh-1 have been also identified to form a p53 negative feedback loop [25]. Later, more ubiquitin ligases are identified to be involved in the regulation of p53 degradation. These include Pirh2, Trim24, and Arf-BP1 among others [26]. Moreover, several other ubiquitin ligases including Topors, Chip, Carp1, Carp2, p300, and CBP are identified to specifically mediate poly-ubiquitination of p53 [27]. It should be also pointed out that among the ubiquitin ligases, Mdm2 plays a central role among all the p53 feedback loops, whereas the other ubiquitin ligases mainly substitute the roles of Mdm2 to complement the deficiency of Mdm2 in Mdm2-deficient cells [25,26].

p53 can be activated by a variety of stimuli including IR, UV radiation, activated oncogenes, hypoxia, and chemotherapeutic drugs. However, our current understanding about the p53-mediated stress response mainly results from the response to double-strand DNA breaks (DSBs) induced by IR and single-strand DNA breaks (SSBs) induced by UV radiation [23,24,28]. The response of p53 to SSBs is mediated by a less-sensitive stress sensor, ATR [28], which activates p53 and promotes degradation of Mdm2, thereby leading to an increased level and activity of p53. Although activated p53 can also upregulate Mdm2 expression, Mdm2 protein degradation is much faster than its protein synthesis. This results in a low level of Mdm2 protein that in turn leads to a sustainable level of p53. This further leads to a prolonged p53 pulse that subsequently activates pro-apoptotic downstream effectors [28]. Although this model is supported by some experimental results, it needs to be confirmed by additional evidence that can illustrate the roles of the ATR-p53-Mdm2 negative feedback loop in mediating the response of p53 to UV radiation. The response of p53 to DSBs is mediated by the ATM-p53-Wip loop [29]. In this loop, IR-induced DSBs are initially detected by ATM, which then activates itself by auto-phosphorylation. Subsequently, Mdm2 is degraded, and p53 protein is stabilized and activated via phosphorylation. Activated p53 then transactivates Wip1, a phosphatase that dephosphorylates activated ATM. This creates a negative feedback loop between ATM and p53 [29,30]. Thus, ATM-dependent phosphorylation prevents Mdm2 from binding and ubiquitinating p53, leading to stabilization of p53 and further transactivation of Mdm2 gene. The feedback loop appears to be involved in the initiation of transient p53 pulses in response to IR because silencing of the *WIP1* gene can result in the dynamics induced by UV-like stimuli [31]. It has been shown that ATM responds to DNA strand breaks fairly efficiently. One or two DSBs in the human genome are sufficient to partially activate ATM, and less than 20 DSBs can lead to full ATM activation [32]. Thus, transient p53 pulses mediated by ATM are highly sensitive to IR stimuli.

**Figure 4:**
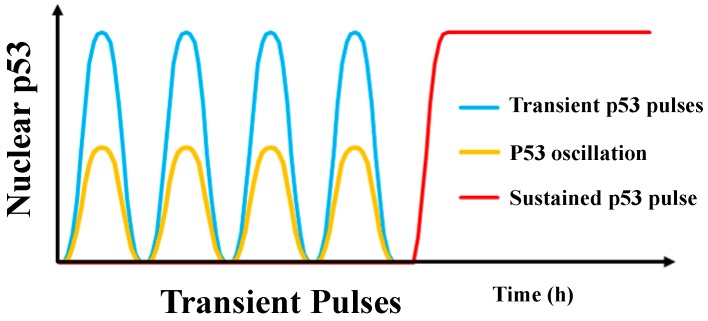
Different dynamic patterns of p53. p53 oscillations (yellow line) that occur at the early stage of DNA damage but with a low amplitude, constitute the basis of p53 dynamic behaviors. Transient p53 pulses (blue line) with a high amplitude occur at the early stage of DNA damage resulting in cell cycle arrest and DNA repair. Sustained p53 pulses (red line) occur at later stages of DNA damage leading to apoptosis.

Because IR-induced p53 pulses are fixed in amplitude and duration, it was initially proposed that the decision of cell fate is governed by the frequency of p53 pulses [31]. It is conceivable that a low dose of IR induces transient p53 pulses with a low frequency, which in turn leads to cell cycle arrest and subsequently to cell survival and recovery. In contrast, a high dose of IR induces a high frequency of p53 pulses, and if the frequency exceeds a threshold, apoptosis occurs. The transient p53 pulse mode can prevent the p53 protein level from being over-activated, allowing cells to recover from DNA damage. Thus, this mode only results in pro-arrest cellular responses [31,33]. However, although the p53 transient pulses can trigger apoptosis, it may still take several hours for the apoptotic process to be initiated after a “death” decision is made. On the other hand, the sustained p53 pulse mode leads to a high level of p53 protein, which should be able to trigger apoptosis much more quickly and efficiently. Therefore, it was later proposed that transient p53 pulses must be orchestrated with sustained p53 pulses to constitute a flexible and efficient regulation of the cell fate decision [34] (Figure 4). However, the mechanisms of how transient p53 pulses can be switched to sustained pulses remain to be elucidated. It has been proposed that some of the p53 positive feedback loops may promote sustained p53 pulses, thereby maintaining a high level of p53, and this efficiently induces apoptosis. Based on this hypothesis, employing mathematical models and experimental data, several hypothetical positive feedback loops for producing sustained p53 pulses through DDR1, DAPK1, PTEN and c-Ha-Ras, have been created [13,34]. In a model proposed by the Wang group [13], a low dose of DNA damage induced by IR at 3 Gy for less than 1500 min, results in the phosphorylation of p53 at the Ser-15 and Ser-20 by the phosphorylase ATM. The partially-phosphorylated p53 subsequently transactivates p21, Wip1, and p53-dependent damage inducible nuclear protein 1 (p53DINP1). p21 then induces cell cycle arrest allowing the damage to be repaired. On the other hand, the phosphatase Wip1 promotes the dephosphorylation of ATM. This suppresses the activity of p53 leading to cell survival. However, if the severity of DNA damage increases continuously, reaching the level equivalent to the one induced by a high dose of IR at 3Gy for longer than 1500 min or IR at 5Gy or above, the phosphorylase, p53DINP1 further phosphorylates p53 at Ser-46, resulting in the formation of fully-phosphorylated p53. The fully-phosphorylated p53 transactivates p53DINP1, p53-regulated apoptosis-inducing protein 1 (p53AIP1) and PTEN. Subsequently, p53AIP1 and PTEN activate the downstream cytochrome C-caspase 3 cascade, thereby inducing apoptosis. However, no experimental evidence has been obtained to show that any of these positive feedback loops play an essential role in prolonging p53 pulses. Instead, a newly identified positive feedback loop, the p53-Rorαloop, has been shown to play a pivotal role in triggering a sustained p53 pulse induced by DNA damage from a wide range of stimuli. This is supported by many experimental observations [35] further indicating that this positive loop is critical in mediating prolonged p53 pulses.

#### 2.2.2. How Does Pulsatile p53 Dynamics Make the Cell Fate Decision?

With the discovery of multiple functional feedback loops that result in different p53 dynamics (transient or sustained pulse), an emerging question is how the p53 dynamics determine cell fate in a coordinated manner? To address this, several computational models that integrate transient and sustained p53 pulses have been developed to understand the cell fate decision process. The models have shown that in response to UV radiation, p53 exhibits a single prolonged pulse that is associated with the irreversible cell fate—apoptosis [28]. In contrast, in response to IR, p53 employs a two-phase response to determine cell fate [13]. In the first phase, the protein level of p53 acts as a series of transient pulses leading to cell cycle arrest and recovery. If IR-induced DNA damage is repaired, p53 dynamics will return to a normal status, and cells will survive. However, if DNA damage remains, p53 dynamics will enter into the second phase during which the dynamics of p53 are switched from a transient pulse mode to a sustained mode with a high level of p53 protein that triggers apoptosis. Thus, the two-phase cell fate decision mechanism mediated by p53 dynamics ensures a flexible and efficient regulation that can avoid a premature apoptosis process as well as initiate the execution of apoptosis.

It is noteworthy that the majority of studies on p53 dynamics focus on how p53 responds to stimuli, i.e., exogenous DNA damaging agents such as IR and UV radiation. Few studies explore how p53 reacts to intrinsic or spontaneous DNA damage caused by normal physiological processes. With quantitative time-lapse microscopy of individual human cells (MCF7 cell line), a recent study showed that p53 exhibits excitable spontaneous transient pulses in non-stressed cells to respond to spontaneous DNA damage with a dynamic pattern similar to that in response to IR [32]. This finding indicates that p53 can also be quickly activated in response to spontaneous DNA damage in cells. This further indicates that the dynamics of p53 protein level as a part of “p53 dynamics” govern cell signaling that mediates cellular responses to both endogenous/spontaneous and exogenous/environmental DNA damage.

## 3. The Dynamics of p53 That Are Independent of Its Cellular Protein Level

In general, p53 dynamics are designated as the variation in p53 protein level in response to stimuli. However, this notion has been challenged by the fact that the maintenance of a steady level of p53 protein induced by IR with a p53-stabilizing agent only can switch cell fate from survival to irreversible senescence [19]. This is different from the UV-induced outcome, apoptosis. This suggests that the dynamics of p53 protein level probably represent only one aspect of p53-mediated cell fate decision. It appears that other aspects of the dynamics of p53, such as their spatial location and post-translational modifications, may also play crucial roles in mediating cellular DNA damage responses by coordinating with the dynamics of its protein level.

### 3.1. The Spatial Dynamics of p53 in Cells

As a transcription factor, p53 is continuously transported among the nucleus, cytosol, and mitochondria. Export of p53 from the nucleus to mitochondria is thought to be a negative regulation of p53 transcription activity by excluding the protein from its target genes. Yet, recent studies have shown that non-transcriptional mitochondrial p53 proteins also play an important role in p53 dynamics at the spatial level of the protein [36,37,38].

Because of its regulatory roles in gene transcription, p53 proteins are mainly located in the nucleus, where they bind to the promoter and/or enhancer of the target genes to regulate gene expression. Yet, a small portion of p53 protein is located in mitochondria to perform its non-transcriptional function. It appears that such a small amount of p53 in mitochondria is sufficient to mediate mitochondrial apoptosis effectively [36,39]. Upon DNA damage, p53 translocates into the mitochondrial outer membrane to interact with Bcl2 family members, Bak/Bax. This promotes Bak/Bax oligomerization, leading to mitochondrial outer membrane permeabilization, cytochrome c release, caspase-3 activation, and apoptosis [37,40,41]. Thus, mitochondrial p53 exhibits a pro-apoptotic activity and acts much faster than nuclear p53. It has been reported that p53 can accumulate in mitochondria within 30 min of stimuli exposure to trigger apoptosis prior to the activation of p53 target pro-apoptotic genes in the nucleus [38].

It appears that nuclear and mitochondrial p53 are orchestrated to determine the fate of cells that are damaged by IR [9]. This has been described in a computational model, where depending on the severity of DNA damage, three types of cellular outcomes would occur: survival, cell cycle arrest, and subsequent and immediate apoptosis without repair. If the damage is severe and can result in full phosphorylation of p53 at Ser-15/20/46, mitochondrial p53 can directly activate apoptosis to eliminate severely damaged cells. If the damage is less severe and can only partially phosphorylate p53 at Ser-15/20, p53 can then accumulate in the nucleus and act as a transcription factor to mediate the “two-phase cell fate decision mechanism” described in Section 2.2. Thus, p53 can save cells that are less severely damaged, but direct cells that are severely damaged to apoptosis.

### 3.2. The Dynamics of Posttranslational Modifications of p53

The posttranslational modifications of p53—including ubiquitinylation, phosphorylation, methylation, and acetylation [42]—play important biological functions. Ubiquitinylation of p53 is mainly mediated by Mdm2 [21]. The post-translational modification of p53 was initially identified in the 1990s as a key regulator of the protein by promoting proteasomal degradation of p53 [22]. Later, multiple types of Mdm2-mediated p53 ubiquitinylation were identified [21,43]. It has been found that polyubiquitinylated p53 with a polymeric ubiquitin chain containing at least four ubiquitin subunits attached to a single lysine residue of the protein, is destined to proteasomal degradation. In contrast, monoubiquitinylated p53, conjugated only with a single ubiquitin moiety attached to one or multiple lysine residues, is stabilized. Moreover, monoubiquitinylated p53 is involved in sub-cellular trafficking including nuclear export and mitochondrial translocation, which facilitate mitochondrial p53-activated apoptosis [43]. These findings suggest that Mdm2-mediated p53 ubiquitinylation is dynamic in nature. In fact, it also has been suggested that a switch between the two types of p53 ubiquitinylation plays an essential role in coordinating two types of p53 dynamics—the varying p53 protein levels and spatial locations—thereby creating a path of spatiotemporal cell fate decision. It should be noted that recent studies have identified a deubiquitination process that can reverse the effects of ubiquitination of p53 and its degradation. This is accomplished by several deubiquitination enzymes including USP7 that deubiquitinates Mdm2 to stabilize p53 [44] as well as USP10 and USP42 that can directly deubiquitinate p53 upon DNA damage [45,46]. Thus, ubiquitination and deubiquitination of p53 may provide an alternative mechanism for regulating and fine-tuning p53 dynamics and cell fate decision.

**Figure 5:**
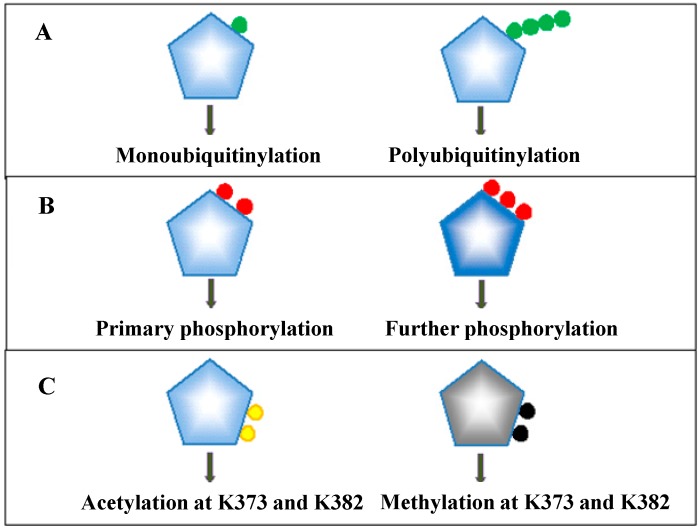
Posttranslational modifications of p53. (**A**) p53 can be ubiquitinylated by Mdm2 in mono- and poly-form at a single site and multiple sites. Polyubiquitinylated p53 can bear a polymeric ubiquitin chain with at least four subunits at a single lysine residue. Green dots indicate ubiquitin units; (**B**) p53 can be phosphorylated at Ser-12/20 facilitating cell cycle arrest. If DNA damage is not repaired, p53 is subsequently phosphorylated at Ser-46 leading to apoptosis. Red dots indicate phosphate groups; (**C**) p53 can be acetylated and methylated at K373 and K382. Yellow dots indicate acetyl groups, whereas black dots indicate methyl groups. The modifications regulate activation and inactivation of p53.

Discovery of the dynamics of p53 ubiquitinylation also led to the discovery of the dynamics of other types of post-translational modifications of p53 (Figure 5). Thus far, there are at least 30 amino acid residues in the p53 protein that have been identified as posttranslational modification sites. Many of the sites can be modified by multiple types of posttranslational modifications that include phosphorylation, acetylation, methylation, and SUMOylation among others (Figure 5). In addition, one type of posttranslational modification can occur on multiple sites of the p53 protein. This unique feature provides a basis for the dynamic changes of various types of p53 modifications at one specific site as well as the dynamics of one specific type of p53 modification at multiple sites. During the process of phosphorylation of p53 for its activation [47], p53 is primarily phosphorylated at Ser-15/20, and then phosphorylated at Ser-46 if stress is sustained [48,49]. The Wang group has developed a computational model to illustrate the biological function of this sequential process of phosphorylation of p53. In this model, it is proposed that the dynamics of p53 phosphorylation can regulate the transcription activity of p53, governing transactivation of p53 target genes selectively [13]. In this scenario, p53 initially exhibits transient pulses in response to IR. This primarily results in phosphorylation of p53 at Ser-12/20, which then partially activates p53 and induces pro-arrest genes for damage repair. If the damage is detected as “the one that cannot be repaired”, p53 is further phosphorylated at Ser-46, and this in turn activates p53 transactivated pro-apoptotic genes leading to the removal of cells containing unrepaired DNA damage via apoptosis.

Acetylation of p53 occurs on several lysine residues at the C-terminal domain of p53 including K370, K372, K373, K381, and K382, and this is carried out by a p53 co-factor, p300/CBP. The modification mainly results in increased DNA binding of p53, promoting the activation of its target gene. In contrast, methylation of p53 by methyltransferases at histidines, arginines, and lysines, suppresses transcriptional activity of p53 [42]. Interestingly, some lysine residues of p53 that are modified by acetylation can also be methylated [50]. However, it has been shown that the two different modifications at K382 of p53 can lead to opposite biological consequences. Acetylation of K382 can result in an increased expression of p21 gene, a target gene of p53 that mediates cell-cycle arrest in cancer cell lines such as MCF7, MCF10, and HCT116. In contrast, methylation of the same lysine residue inactivates p21 in the cell lines. This indicates a distinct role of acetylation and methylation in regulating p53 activity [50]. Furthermore, a recent study reveals that a switch between acetylation and methylation of p53 provides a way for cells to distinguish p53 pulses induced by intrinsic/endogenous damage or by extraneous/exogenous damage [32,50]. It has been shown that in the absence of exogenous stimulation, spontaneous/endogenous stress can induce deacetylation and methylation at K373 and K382 of p53, and this suppresses the transcriptional activity of p53, and the resulting p21 gene expression. This allows p53 to adopt a mode of responding to spontaneous/endogenous damage [32]. In the presence of exogenous stimuli such as 10 Gy of IR or 2 μM adriamycin, a large amount of acetylated p53 can be recruited at the p21 promoter region in cells. This indicates that the transcriptional activity of p53 is stimulated by the exogenous stimuli via acetylation. Interestingly, adriamycin–induced accumulation of acetylated p53 on the p21 promoter can be further stimulated by methylation at K372 of p53 by Set-7/9, a lysine-specific histone methyltransferase [50]. Thus, methylation and acetylation at different lysine residues of p53 and their interplay govern cellular response to spontaneous/endogenous and exogenous damage, respectively.

### 3.3. Roles of MicroRNAs (miRNAs) in Modulating p53 Dynamics

Recent studies have shown that besides its crucial role in regulating gene expression, p53 can also activate its target miRNAs, which include miR-192, miR-194, miR-215, miR-107, and miR-605 [51,52]. Interestingly, among the miRNAs, miR-605, miR-143, and miR-145 induced by p53 can also enhance p53 activation by suppressing the translation of Mdm2 mRNA and its protein synthesis [53]. On the other hand, p53-induced miR-16 and miR-34a can promote p53-induced apoptosis by suppressing the expression of an anti-apoptotic protein, Bcl-2 [54], an anti-apoptotic protein that can inhibit p53-regulated apoptosis-inducing protein 1 (p53AIP1) by forming a Bcl2-p53AIP protein complex [55]. The roles of miRNAs in modulating p53 dynamics have been illustrated in a computational model that describes the roles of miR-605 and miR-34a in IR-induced p53 dynamics [56]. In this model, IR-induced DSBs activate DNA damage sensor ATM that in turn activates p53. Activated p53 subsequently upregulates the expression of Mdm2 and miR-605, which in turn suppresses the translation of Mdm2 mRNA, thereby promoting the activity of p53 [57]. Thus, p53, miR-605, and Mdm2 constitute a positive feedback loop that facilitates a series of transient pulses resulting in cell cycle arrest and recovery. On the other hand, miR-34a contributes an additional effect to this positive feedback loop by suppressing the expression of Bcl-2 upon severe DNA damage that cannot be repaired by cells. This then allows release of cytochrome C and initiation of caspase-3 cascade for apoptosis [58]. This model demonstrates that p53-targeted miRNAs play crucial roles in modulating p53 dynamics and signaling network.

### 3.4. The Network of p53 Dynamics in Governing Cell Fate and Cancer Therapy 

**Figure 6:**
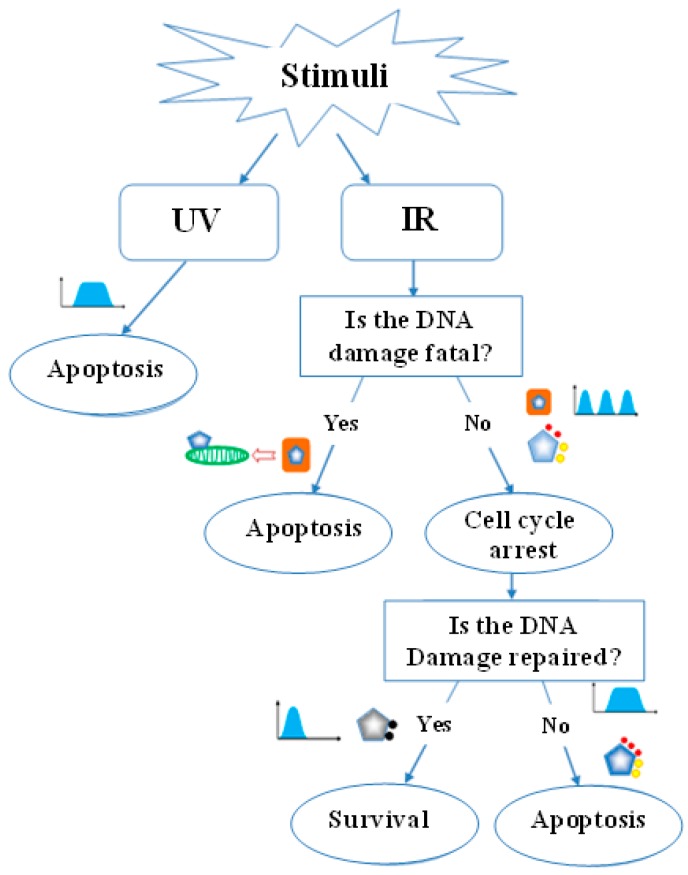
p53 dynamics in the cell fate decision. Cells under the attack of different types of DNA damage can be directed to different fates. UV-induced DNA SSBs result in a single prolonged pulse of p53 directing cells to the path of irreversible apoptosis. IR-induced DSBs can result in different consequences depending on the extent of DNA damage. In responding to DSBs, cells evaluate the severity of damage repeatedly. If damage is too severe to be repaired, Mdm2 mediates mono-ubiquitinylation of p53 initiating its nuclear export and accumulation in mitochondria. This then initiates the transcription-independent apoptotic program. If DNA damage is repairable, p53 in the nucleus serves as a transcription factor to function in a pulsatile dynamic manner at its protein level. Initially, the p53 protein level exhibits a series of transient pulses, which in turn promote primary modifications of p53 including phosphorylation at Ser-15/20, and acetylation/ demethylation at K373 and K382. These partially activate p53 subsequently transactivating pro-arrest genes and induce cell cycle arrest. Then cells determine whether DNA damage is fixed or not. If it is fixed, p53 returns to its inactive form, and cells survive. If DNA damage cannot be fixed, transient p53 pulses are switched to sustained pulses leading to full activation of p53 through further posttranslational modifications, such as the addition of phosphorylation at Ser-46 and ultimately resulting in apoptosis.

Our current understanding of p53 dynamics is limited to the pulsatile switches of its protein level. However, the interplay among different types of p53 dynamics is critical in governing a specific cellular damage response. Based on this, p53 dynamics can be designated as a multi-faceted dynamic network comprised of all variables that can be measured over time. These include p53 protein level, its activity, subcellular localization and posttranslational modifications. We therefore propose a model that illustrates the roles of multiple dynamic features of p53 in governing cell fate (Figure 6). In non-stressed cells, p53 exhibits spontaneous transient pulses that are usually triggered by endogenous DNA damage. In this scenario, because damage signals are not sustainable, the posttranslational modifications of p53, including deacetylation and methylation at residues K373 and K382, result in inactivation of p53 protein, thereby preventing it from inducing cell cycle arrest. In cells that are attacked by stressors, such as UV radiation, oncogene activation, and IR, continuous production of DNA damage, including SSBs or DSBs, can activate p53 transcription activity. In response to UV radiation and activated oncogenes, p53 exhibits a single prolonged pulse that subsequently leads to an irreversible apoptotic process. In response to IR-induced DNA damage, p53 regulates cell fate by causing three different types of consequences—i.e., survival, apoptosis after damage repair, and immediate apoptosis. Thus, when IR-induced damage occurs, it is repeatedly evaluated by cells for determining their fates. If the damage is too severe to be repaired, p53 will translocate from the nucleus to mitochondria. This will rapidly trigger a transcription-independent apoptotic process eliminating severely damaged cells without consuming energy. If the damage is repairable, p53 will stay in the nucleus and act as a transcription factor. In this scenario, p53 will exert a series of transient pulses promoting its primary modifications such as acetylation and demethylation at K373 and K382, and subsequent phosphorylation at Ser-15/20. This will partially activate p53, resulting in upregulation of pro-arrest gene expression that in turn facilitates damage repair. Cells will then determine whether the damage is sufficiently repaired or not. If the damage is fully repaired, p53 will return to an inactive status, and cells will survive. Otherwise, p53 pulses will switch from a transient to a sustained mode. This will then initiate additional posttranslational modifications of p53, mainly phosphorylation at Ser-46. This will completely activate p53 and ultimately activate pro-apoptotic gene expression and apoptosis.

The central role of p53 in governing the cell fate decision and preventing tumorigenesis makes it a promising target for cancer therapy. Over the past decades, p53-based cancer therapeutic strategies have mainly focused on p53 gene-transfer therapy with an adenovirus vector, or on restoring p53 activity using small molecule drugs that interact with mutant p53 protein and alter the conformation of the protein [59]. These therapeutic approaches increase the total level of functional p53 protein in tumor cells. However, manipulation of multiple p53 dynamic profiles in a controlled manner may lead to a more effective cancer therapy. Recent studies have shown that small molecules, Nutlin and RITA, which can disrupt p53-Mdm2 binding may be used to stabilize the p53 protein level. These two compounds are recommended be used in the clinics for treatment of tumors with Mdm2 overexpression [60]. Thus, p53 dynamics-based therapy can be developed as a new strategy for cancer treatment. 

However, it should be noted that such cancer therapeutic strategies may be complicated by the multiple roles of Mdm2. This is because Mdm2 can mediate translocation of p53 from the nucleus to mitochondria to initiate rapid apoptosis [36,37]. This indicates that Mdm2 can act as both an oncoprotein and a critical protein that mediates apoptosis. Thus, the current Mdm2-based cancer therapy, which solely focuses on inhibition of Mdm2, may have its limitations. A dual role of Mdm2 in serving as both an oncoprotein and a tumor suppressor should be considered in an effort to improve anticancer therapeutic efficacy. Based on this, a Mdm2-based cancer therapy can be achieved by two approaches: (1) Since Mdm2 mediated mono-ubiquitinylation of p53 is the key for p53 spatial trafficking and subsequent apoptosis initiation, inhibition of polyubiquitinylation of p53 can lead to an effective therapy. Some compounds that can deubiquitinylate p53 such as USP10 may be used as a potential therapy [61]; (2) Based on the fact that the competition between nuclear p53 and mitochondrial p53 governs the non-transcription dependent apoptosis, inhibitors of nuclear p53 can be used to enhance the mitochondrial p53-triggered rapid apoptosis process, thereby increasing the efficacy of chemotherapeutics.

In a scenario where the mitochondrial p53-mediated apoptotic pathway fails to be initiated, a nuclear p53-mediated apoptosis can be initiated as an alternative pathway to promote cell death. A critical step of this process is to promote p53 transactivation of pro-apoptosis genes. Based on the fact that p53 transcriptional activity is determined by both its protein level and posttranslational modifications, two strategies can be employed to alter p53 transcriptional activity. One is to switch p53 pulses from a transient mode to a sustained mode. Defects in this switch have been identified in human cancers in a tissue-specific manner. For example, Wip1, a phosphatase, is essential for the initiation of transient p53 pulses. Thus, it acts as an oncogene in many cancers such as breast cancer, glioma, and colon carcinoma [62]. In contrast, PTEN, a tumor suppressor that can induce a sustained p53 pulse, is not expressed in MCF-7 human breast cancer cells [13]. Thus, inhibition of Wip1 or stimulation of PTEN can alter the p53 dynamic pattern, thereby facilitating p53-dependent apoptosis and improving cancer treatment efficacy in a tissue-specific manner. The other strategy is to switch p53 phosphorylation from partial to full. This can be achieved by altering a hub within the p53 phosphorylation process, stimulating p53 phosphorylases such as DYRK or inhibiting phosphatases such as Wip1. It should also be noted that the cellular level of p53 appears to be the key that underlies p53 transcriptional activity, yet it is delicately regulated by the posttranslational modifications of the protein resulting in a specific pattern of p53 dynamics in cells. This provides a variety of targets that can be modulated for regulating cellular level of p53 and manipulating cell fate. However, the multiple dynamic features of p53 are also challenges to the current p53-based cancer therapeutic strategies. In addition, the effects of p53 mutants on p53 dynamics can modulate the efficacy of p53-based cancer therapy. It has been found that in tumor cells, most p53 mutant proteins bear a surface cleft that mediates its physical interaction with Mdm2, thereby leading to p53 degradation [63,64]. It appears that for the treatment of such a type of tumors, a therapy that increases the p53 level by extending the half-life of p53 should be a priority before a p53 dynamics-based therapy can be considered. This can be achieved by p53 gene-transfer or p53 activator drugs, such as Prima-1(Met)/Apr-246 and phenethyl isothiocyanate (PEITC) so that the cellular level and activity of p53 may be restored to normal [64,65]. Thus, different strategies may be adopted for p53-based cancer therapy upon the different types of p53 deficiency in tumor cells.

## 4. Conclusions

The dynamics of p53 have emerged recently as a new field of cell signaling. However, its biological significance remains to be elucidated. With the development of fluorescent labeling and time-lapse technology, studies on p53 dynamical profiles in protein level, spatial location, and posttranslational modifications will provide new insights into the biological function and implications of p53 dynamics in cancer treatment. Because p53 dynamics have been mainly studied in IR-induced DNA damage models, many of which are based on computational and mathematic approaches, additional experimental results that support these models are in urgent need. These include the results of p53 dynamics induced by other stimuli such as ROS, heavy metals, and chemotherapeutic drugs. In addition, because the measurements of p53 dynamics are currently restricted in cultured cells, the dynamic profiles of this protein need to be further verified in a biological system such as fruit flies, worms, zebra fish, mice, and humans, if it is feasible. Nevertheless, our knowledge about dynamic interactions among various cell signaling networks and the dynamics of the components of the network will provide valuable guidance to manipulate and control cell signaling pathways for achieving therapeutic purposes in both cancer and other human diseases such as neurodegenerative diseases.
